# Field-Based Evaluation of Insecticide Effectiveness on *Megalurothrips usitatus* in Guangdong, China: Implications for Pest Control Strategies

**DOI:** 10.3390/insects16050459

**Published:** 2025-04-27

**Authors:** Zhengke Peng, Mengni Li, Chaosong Guo, Huixin Zheng, Mingyue Wu, Fei Yin, Yong Xiao, Huanhuan Wang, Xiangyi Kong, Myron P. Zalucki, Wen Xie, Zhenyu Li

**Affiliations:** 1 Institute of Plant Protection, Guangdong Academy of Agricultural Sciences, Key Laboratory of Green Prevention and Control on Fruits and Vegetables in South China Ministry of Agriculture and Rural Affairs, Guangdong Provincial Key Laboratory of High Technology for Plant Protection, Guangzhou 510640, China; zkpeng0827@163.com (Z.P.); lininim@163.com (M.L.); guo.cs@foxmail.com (C.G.); feier0808@163.com (F.Y.); xiaoyong@gdaas.cn (Y.X.); 18763823509@163.com (H.W.); 2 Guangdong Provincial Key Laboratory of Insect Developmental Biology and Applied Technology, Institute of Insect Science and Technology, School of Life Sciences, South China Normal University, Guangzhou 510631, China; 3 Key Laboratory of Green Control of Crop Pests in Hunan Higher Education, Hunan University of Humanities, Science and Technology, Loudi 417000, China; zhenghx0124@163.com; 4 Sanya Academy of Tropical Agricultural Sciences, Sanya 572000, China; wumingyuecaas2021@163.com (M.W.); kongxiangyi20@163.com (X.K.); 5 School of the Environment, The University of Queensland, St. Lucia, QLD 4072, Australia; m.zalucki@uq.edu.au; 6 State Key Laboratory of Vegetable Biobreeding, Institute of Vegetables and Flowers, Chinese Academy of Agricultural Sciences, Beijing 100081, China; xiewen@caas.cn; 7 Sanya National Academy of Southern Propagation, Chinese Academy of Agricultural Sciences, Sanya 572019, China

**Keywords:** cowpea, bean flower thrips, insecticide toxicity monitoring, resistance management, IPM

## Abstract

In southern China, cowpea production is severely threatened by *Megalurothrips usitatus* due to its fast-evolving resistance to insecticides. The toxicity monitoring of commonly used insecticides against field populations provides key information for the resistance management of pests. In this study, field populations of *Megalurothrips usitatus* were collected from three locations (QY, YF, MM) in Guangdong, and the sensitivity of these populations against insecticides was determined by using a thrips insecticides bioassay system (TIBS) method. The bioassay results indicated there were sensitivity variances to insecticides between these three field populations. Among these 10 insecticides, spinetoram and spinosad both showed high toxicity against all three field populations. In addition, broflanilide for QY, emamectin benzoate for YF, and emamectin benzoate and cyantraniliprole for MM were suggested as alternate insecticides to alleviate selective pressure from insecticides on field populations. In field experiments, the corrected control efficacy of cyantraniliprole and spinetoram against *M. usitatus* was over 75% at 7 dpa, which proved to be ideal insecticides for field application. These field-based results provide guidance for chemical control against thrips and can be valuable in proposing appropriate strategies for thrips resistance management.

## Simple Summary

This study evaluated the toxicity of several commonly used insecticides against cowpea thrips in Guangdong, which provided comprehensive data for the rational choice of insecticides and advice for the integrated management of thrips.

## 1. Introduction

Cowpea (*Vigna unguiculate* [L.] Walp.) was domesticated in Africa, while its cultivation has been distributed throughout most of the globe due to its inherent resilience to harsh conditions like drought and high temperatures [1,2]. Additionally, as a dependable source of plant-based protein and folic acid, cowpea is often regarded as a safe food integrated into sustainable agriculture systems in the context of global climate change [3]. Being among the top ten cultivated vegetables in Asia, cowpea is planted across China, with a cultivation area of more than 0.67 million ha per year and annual production of 1.5 million tons [4,5,6]. In southern China, especially Hainan and Guangdong Provinces, where climate conditions are still suitable for the cultivation of vegetables in winter, cowpea supplied to the northern areas constitutes the main income for local growers due to its high economic value and production. However, both the quality and production of cowpea are seriously hindered by thrips, with an 80% reduction in cowpea yield or complete crop failure in some severe cases [7].

*Megalurothrips usitatus* (Bagnall) (Thysanoptera: Thripidae) is the dominant thrips species in most cowpea-producing fields in China, which can damage all developmental stages [8]. Thrips steal nutrition from organs like the leaves, flowers and pods of cowpea by using their specialized rasping–sucking mouthpart, causing leaf wrinkling, growth point atrophy, necrosis, premature bud and flower drop, and pod scab, along with black-heads and black-tail symptoms, which impairs cowpea and leads to severe economic losses [9,10]. Thrips are sophisticated at concealing themselves in those apertures of the cowpea plant by virtue of their tiny bodies and positive thigmotaxis nature [11,12,13], making them formidable to control. Strategies of integrated pest management (IPM) concerning thrips like *M. usitatus* are widely applied in the field, including agronomic control, physical control, and biological control, as well as chemical control, among which chemical control is currently the most effective. However, their high reproductive capacity and rapidly developed resistance against insecticides considerably challenge the field management of thrips, and the indiscriminate use of insecticides directly leads to “poisoned pods” events, arousing public concern about food safety [4,14,15,16].

Bioassays indicated that *M. usitatus* has developed resistance to neonicotinoids, and the pyrethroids and molecular mechanisms underlying neonicotinoid resistance in thrips have been identified [17,18]. With the high selective pressure in the field, the fast-evolved resistance of thrips to other kinds of insecticides can occur, making the monitoring of field resistance critical for the rational choice of insecticides. The previous toxicity monitoring of insecticides against *M. usitatus* was mainly focused on field populations in Hainan [19,20], while there are few data available for Guangdong, where the usage of insecticides and cropping systems differs considerably from those in Hainan. In contrast to Hainan Province, cropping systems of mixed plantings of cowpea and towel gourd are typical, and overall insecticide use is not as intensive in Guangdong Province, which is responsible for the likely variance in the resistance level of *M. usitatus* against insecticides [21,22].

Therefore, more area-specific data of insecticide toxicity against cowpea thrips are a requisite for the design of strategies that alleviate resistance development. In this study, ten commonly used insecticides representing different modes of action were selected, including macrolides, spinosyns, diamides, neonicotinoids, ketoenols as well as pyrroles. Field populations of *M. usitatus* were collected from Qingyuan (QY), Yunfu (YF) and Maoming (MM), being located from the east to west of Guangdong Province (Figure S1). These three locations are widely separated and among the main cowpea-growing areas in Guangdong. Apart from laboratory toxicity monitoring, five key insecticides, which are regularly used in these locations, were selected for further evaluation of field control efficacy against *M. usitatus.* These area-specific toxicity data will help to make informed decisions when selecting insecticides for the control of thrips.

## 2. Materials and Methods

### 2.1. Field Populations

Field populations of *M. usitatus* were collected from three areas, Maoming (MM), Yunfu (YF), and Qingyuan (QY) in Guangdong, in 2023 (Figure S1). Thrips were collected from cowpea plants in their full-bloom stage. All collected thrips from each location were maintained in transparent tissue culture bottles with top lid cut and a 200-mesh screen covered, as previously described [16]. Newly emerged adults of the F1 generation of thrips were used for the toxicity bioassays.

### 2.2. Insecticides

Ten insecticides, which are commonly used in the field of Guangdong, were selected, namely broflanilide 100 g·L^−1^ SC (BASF, Ludwigshafen, Germany), dinotefuran 20% SC (Qingdao Haina biotechnology limited company, Qingdao, China), spinetoram 60 g·L^−1^ SC (Dow AgroSciences, Indianapolis, America), spinosad 10% SC (Deqiang biotechnology limited company, Harbin, China), cyantraniliprole 10% OD (FMC, Philadelphia, America), spirotetramat 22.4% SC (Bayer crop science, Leverkusen, Germany), emamectin benzoate 5% ME (Shenzhen Noposion crop science, Shenzhen, China), avermectin 5% EC (Zhengbang crop, Nanchang, China), thiamethoxam 25% WG (Syngenta Group, Shanghai, China), chlorfenapyr 100 g·L^−1^ SC (NPS crop science, Shenzhen, China). The recommended dosages of insecticides are indicated in Table S1.

### 2.3. Bioassays for Insecticide Toxicity

The toxicity bioassay method was modified from TIBS, as previously described [19]: using a modified 1.5 mL centrifuge tube cut at a slant at the bottom, with a small hole of about 0.5 cm in diameter on the cap of the tube covered with 200-mesh gauze. Insecticides were diluted into five~seven concentration gradients (Tables S2–S4) via the doubling method, four replicates (assay tubes) were set up for each concentration gradient, and about 15 healthy female thrips were used in each replicate. For each replicate, thrips were fed with one bean pod, which was immersed in insecticide solutions or water control for 15 s. After 48 h, the survival conditions were observed and recorded.

### 2.4. Field Experiment

We selected five insecticides commonly used in the field and carried out field experiments in a farm located in Yunfu city. Each treatment was assessed using four replicate samples, and each replicate included 15 flowers taken off from the top, middle and bottom of randomly chosen cowpea plants in each replicate plot. These flowers were collected into a ziplock bag, which were afterwards put into hot water for several minutes to let thrips come out from the flowers. Then, 75% methanol was sprayed into the ziplock bag by using a spray bottle to kill remaining thrips. After then, thrips and flowers in the bag were rinsed with water and poured out into a bottom white tray. Flowers in the tray were removed after the rinsing. Pictures were taken for each tray in order to count the number of thrips.

Insecticide recommended doses as the producers suggested were applied in the field experiment, with water spay as a mock control. To ensure insecticides were evenly sprayed onto the whole plant, a manual air-pressure backpack sprayer with a yellow hollow cone nozzle was used, and the working pressure was 0.2–0.3 Mpa. Cowpea was planted in twin rows and was under the same cultural conditions and well managed in every plot, including standardized irrigation and fertilizer application regimes throughout the growing season. Each row included a 0.4 m aisle, 0.25 m between plants in a row, and 1.0 m between plants in a pair. Each plot included a sample (treated) zone, surrounded by buffer zone, which consisted of untreated cowpea planted in the margin of each plot (Figure S2). A total of 24 plots were used in this study, allowing for six treatments (five treated with insecticide spray plus one water spray as control) with four biological replications. Specific treatments for each plot were arbitrarily assigned by a random number generator (Excel 2016, Microsoft, USA). The spraying of insecticides was conducted before 10 a.m. when flowers remained open, and cowpea plants were in a prime flowering period.

### 2.5. Data Analysis

The raw data from the bioassays were recorded and used to calculate median lethal concentration (LC_50_) values and 95% confidence limits based on probit regressions with POLO plus 2.0 software (LeOra Software, Berkeley, CA, USA). Toxicity difference ratio (TDR) = LC_50_ of QY population (or YF population)/LC_50_ of MM population. Pictures of different developmental stages of *M. usitatus* were taken under a digital Keyence VHX-6000 microscope.

All statistical analyses were performed with GraphPad Prism (v.8.3.0, GraphPad Software Inc., La Jolla, CA, USA). Prior to analysis, population reduction rate and corrected control efficiency were calculated as the following formula: population reduction rate (%) = (initial number of thrips − number of thrips after treatment)/initial number of thrips × 100% [23] and corrected control efficacy (%) = (reduction rate of treatment plot − reduction rate of mock control)/(1 − reduction rate of mock control) × 100% [23]. The corrected control efficiency values were analyzed using one-way ANOVA with Tukey’s HSD test for multiple comparisons.

## 3. Results

### 3.1. Morphological Characteristics of M. usitatus

**Figure 1:**
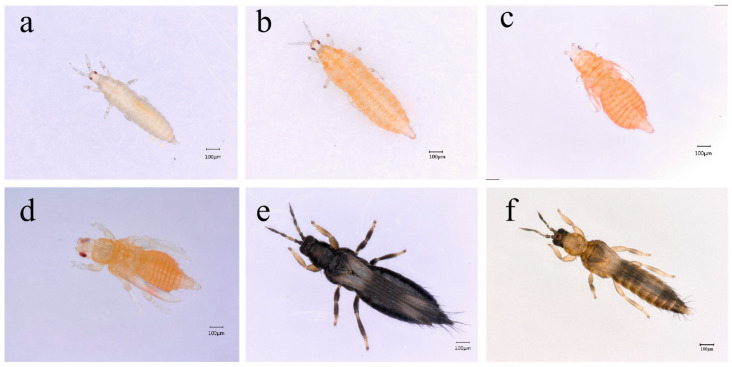
Post-embryonic stages of *M. usitatus*: 1st instar (**a**) and 2nd instar (**b**) are the actively feeding larvae stages. (**c**) Indicates prepupal stage and (**d**) is the pupal stage. (**e**) Female adult and (**f**) is male adult. Scale bars in each picture indicates 100 μm.

The post-embryonic development of *M. usitatus* lies in between the hemimetabolous and holometabolous types, including actively feeding larval stages, inactive prepupal and pupal stages and an adult stage [13]. Newly hatched 1st instar larvae are white (Figure 1a) and turn salmon pink in the 2nd instar (Figure 1b), which undergoes a propupal stage (Figure 1c). Thrips are capable of moving but do not feed in their propupal stage. After several days of development, the insect drops to the soil and enters the pupal stage with wing buds reaching to 2/3 of its abdomen (Figure 1d). Adult females are about 1.5 mm in length, brownish in color with two distinct white bands on the forewing (Figure 1e). Males are smaller in size than females but with barely a difference in color (Figure 1f).

### 3.2. Toxicity of 10 Insecticides Against QY Population

Among the 10 insecticides, spinetoram and spinosad had the highest toxicity against the QY population, with an LC_50_ value of less than 0.5 mg a.i./L; spirotetramat had the lowest toxicity, with an LC_50_ of 987.80 mg a.i./L; broflanilide also showed good toxicity to the QY population, with an LC_50_ of 7.40 mg a.i./L; and emamectin benzoate, chlorfenapyr and avermectin corresponded to LC_50_ values less than 50 mg a.i./L. Dinotefuran, cyantraniliprole and thiamethoxam all exceeded 50 mg a.i./L, with relatively poor toxicity (Table 1).

### 3.3. Toxicity of 10 Insecticides Against YF Population

The toxicity of 10 insecticides against the YF population varied a lot. Emamectin benzoate and spinetoram showed the highest toxicity, and their LC_50_ values were less than 1 mg a.i./L; spinosad corresponded to an LC_50_ of 2.03 mg a.i./L, which also showed high toxicity. In contrast, thiamethoxam, dinotefuran, and chlorfenapyr corresponded to LC_50_ values of about 100 mg a.i./L; spirotetramat and avermectin were the least toxic to YF populations, and their LC_50_ values were 1237.15 mg a.i./L and 675.73 mg a.i./L, respectively (Table 2).

### 3.4. Toxicity of Tested Insecticides Against MM Population

Among the 10 insecticides, avermectin showed the lowest toxicity to the MM population, with an LC_50_ of 360.80 mg a.i./L; spinetoram and spinosad showed the highest toxicity to the MM population, with LC_50_ values of less than 0.5 mg a.i./L; emamectin benzoate corresponded to an LC_50_ of 4.82 mg a.i./L, while cyantraniliprole, dinotefuran, thiamethoxam and chlorfenapyr had LC_50_ of less than 20 mg a.i./L, which indicates their good toxicity against the MM population. The LC_50_ values of broflanilide and spirotetramat against the MM population were 71.33 mg a.i./L and 113.19 mg a.i./L, respectively (Table 3).

### 3.5. Variances in Toxicity of Insecticides Against Different Field Populations

**Figure 2:**
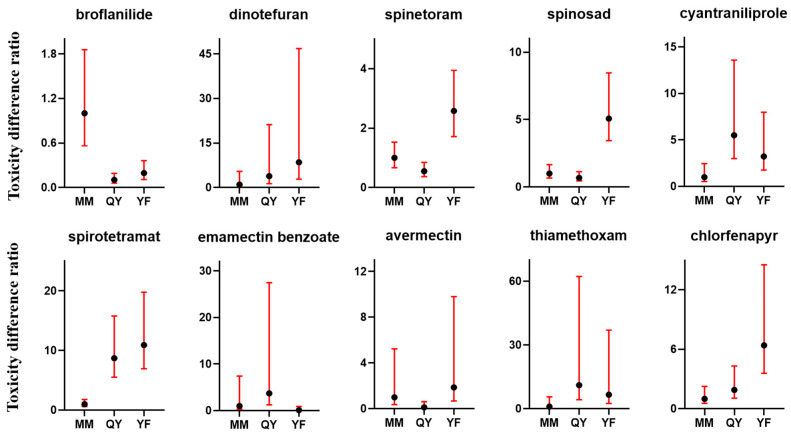
Toxicity difference ratio (TDR) analysis of insecticide against different field strains. Bars of each column indicates upper limits and lower limits of TDR.

There were obvious differences in the toxicity of insecticides against different field populations (Figure 2). The TDR of insecticide against field populations was calculated using the MM population as a reference. The toxicity of broflanilide against the MM population was lower than that against the YF and QY populations (TDR_YF_ = 0.20, TDR_QY_ = 0.10); dinotefuran (TDR_YF_ = 8.52, TDR_QY_ = 3.85), cyantraniliprole (TDR_YF_ = 3.24, TDR_QY_ = 5.51), spirotetramat (TDR_YF_ = 10.93, TDR_QY_ = 8.73), thiamethoxam (TDR_YF_ = 6.61, TDR_QY_ = 11.11), and chlorfenapyr (TDR_YF_ = 6.41, TDR_QY_ = 1.91) were more toxic to the MM population than that to the YF and QY populations. As for the three field populations, spinetoram (TDR_YF_ = 2.58, TDR_QY_ = 0.55), spinosad (TDR_YF_ = 5.07, TDR_QY_ = 0.68), and avermectin (TDR_YF_ = 1.87, TDR_QY_ = 0.12) were the most toxic to the QY population and the least toxic to the YF populations, while emamectin benzoate (TDR_YF_ = 0.12, TDR_QY_ = 3.71) was the most toxic to the YF population and the least toxic to the QY population.

### 3.6. Resistance of M. usitatus to Six Insecticides

There were also variances in the resistance levels of different field populations against several insecticides when compared to the LC_50_ values of susceptible strains reported in the literature [24,25]. For avermectin, the resistance levels of QY, YF and MM populations all reached very high levels, with RR values ranging from 156.92 to 2448.30 (Table 4). For spinetoram, resistance levels showed large differences among various populations; resistance was low for the QY population (RR = 8.74), moderate for the MM population (RR = 15.53) and high for the YF population (RR = 39.47) (Table 4). The QY and MM populations developed very high resistance to emamectin benzoate, while the YF population showed high resistance, with RR values of 2978.33, 802.67 and 98.50, respectively (Table 4). The QY and MM populations developed low resistance to spinosad, while the YF population showed moderate resistance, with RR values of 1.59, 2.30 and 11.80, respectively (Table 4). For spirotetramat, the YF and QY populations obtained very high resistance, with RR values of 939.62 and 314.29, while the resistance of the MM population was high, with an RR of 36.01. For chlorfenapyr, populations QY and YF achieved very high resistance, with RR values of 135.63 and 456.27, respectively. The MM population also developed high resistance to chlorfenapyr (Table 4).

### 3.7. Field Efficacy Trial of Five Selected Insecticides

As shown in Table 5, the corrected control efficacy of the five selected insecticides against field *M. usitatus* was 30~53% at 1 dpa (days post application), 41~60% at 3 dpa, 54~61% at 5 dpa, and 58~77% at 7 dpa. With time increasing, the control efficacy of these five insecticides increased and reached the highest efficacy at 7 dpa, among which cyantraniliprole and spinetoram exceeded to 76.35% and 75.23%, respectively.

## 4. Discussion

Chemical control remains the primary strategy for thrips management in the field, with pyrethroids, neonicotinoids, spinosyns, and avermectins extensively applied against *M. usitatus* over the past decade [16]. However, resistance in *M. usitatus* has escalated annually, driven by the species’ intrinsic traits (positive thigmotaxis, tiny size and high fecundity), combined with intense selection pressure from insecticides [15,19,20]. This necessitates the development of resistance management strategies to ensure sustainable control of *M. usitatus*. Monitoring the resistance levels of field populations provides guidance for the scientific use of insecticides, which also plays a critical role in establishing strategies that delay the development of pest resistance [26]. In this study, we evaluated the toxicity variations in ten widely used insecticides against cowpea thrips sampled across Guangdong. As indicated by the results of laboratory and field experiments, spinetoram remains a useful insecticide for *M. usitatus* control, and insecticides with different action modes, like cyantraniliprole, emamectin benzoate as well as broflanilide, could be integrated into the insecticide rotation system.

The spinosyns are fermentation-derived insecticides acting as allosteric activators of nicotinic acetylcholine receptors (nAChR) and are of potent activity with lower environment effects [27,28]. In this study, two spinosyns were selected for toxicity bioassays, spinetoram and spinosad, which were of high efficacy for the thysanopterans (thrips) control [14,15,28]. The QY and MM populations with spinetoram showed the highest toxicity, and the YF population with spinetoram showed the second highest toxicity (Table 1, Table 2 and Table 3); the LC_50_ values ranged from 0.2 to 0.7 mg a.i./L, confirming the sustained efficacy of spinetoram against field populations in Guangdong despite prolonged usage. Spinosad also showed good toxicity against QY, YF and MM populations, with LC_50_ ranging from 0.27 to 2.03 mg a.i./L (Table 1, Table 2 and Table 3). However, the rapid development of resistance to spinosyns has already aroused concern in thrips control in the USA, Spain and China [29,30,31]. Additionally, a very high resistance level against spinosyns was also found in other insects, such as Colorado potato beetle and tomato pinworm [32,33]. Compared to a baseline (0.017 mg a.i./L) susceptibility of *M. usitatus* to spinetoram, the resistance levels of Guangdong populations were similar to that from Hainan: populations QY and Haikou developed low tolerance; populations MM, Chengmai, Sanya developed moderate tolerance; populations YF and Ledong developed high tolerance [25]. The judicious use of spinosyns through effective resistance management should be taken into consideration for the restoration of susceptibility in *M. usitatus*.

Emamectin benzoate and avermectin are in the avermectin class of insecticides, which are widely used to control a variety of important pests [34]. Previous monitoring suggested that both emamectin benzoate and avermectin showed good toxicity against *M. usitatus* populations in Hainan, with LC_50_ ranging from 0.103 to 0.535 mg a.i./L and 10.969 to 93.008 mg a.i./L, respectively [25]. However, avermectin was no longer effective for cowpea thrips control in Guangdong, especially for YF and MM populations, which all achieved very high resistance when compared to a susceptible baseline (0.276 mg a.i./L). The wide spectrum of effectiveness makes avermectins popular in Guangdong for the control of pests such as whiteflies, worms and spider mites, which may account for the high resistance of *M. usitatus* to avermectins in these areas due to high selective pressure. Hence, broad-spectrum insecticides should be rotated with other highly selective ones and be used sparingly. As a modified version of avermetin, emamectin benzoate performed better; the LC_50_ values of field populations ranged from 0.59 to 17.87 mg a.i./L in Guangdong, although considerable variances in susceptibility among populations were also observed (Figure 2).

Broflanilide and cyantraniliprole are bis-amide insecticides but divided into two groups for their different mechanisms of action, which are reported to be effective in controlling many species of thrips [17,35,36]. Broflanilide functions as a γ-aminobutyric acid-gated chloride channel negative allosteric regulator while, cyantraniliprole acts as ryanodine receptor modulators [35,37]. Populations YF and QY were more susceptible to broflanilide than cyantraniliprole, while the susceptibility of the MM population to these insecticides was just the opposite. The differential usage of broflanilide and cyantraniliprole in these regions may explain the variances in susceptibility. In addition, when compared to a population from Hainan with an LC_50_ of 2.28 mg a.i./L [17], populations in Guangdong had already developed low to high resistance to broflanilide, which also indicates the issue of considering intraspecific differences in susceptibility when using broflanilide in the field.

Neonicotinoids are currently the most widely used insecticides for their high agonist effects on insect neuronal nicotinic acetylcholine receptors (nAChRs), which are usually preferred for the control of piercing–sucking pests, such as thrips [38,39]. The reduced susceptibility of western flower thrips against thiamethoxam has been found [36], while no such resistance has been reported in bean flower thrips, probably due to a lack of baseline susceptibility. The values of LC_50_ for the QY and YF populations against thiamethoxam were over 100 mg a.i./L (Table 1 and Table 2), which were more than five-times higher compared to the value of a population in Haikou [19]. Other neonicotinoids, like imidacloprid and acetamiprid, showed decreased toxicity against *M. usitatus* [25]. However, thiamethoxam and dinotefuran maintained relatively good toxicity to the MM population with an LC_50_ of around 15 mg a.i./L (Table 3), which suggested differences in susceptibility among field populations in Guangdong.

Chlorfenapyr is a halogenated pyrrole, and spirotetramat is a member of the ketoenol family, which has totally different modes of action [40,41]. However, the very high resistance of the QY and YF populations developed for both insecticides (Table 4). It is, therefore, necessary to rotate chlorfenapyr and spirotetramat with other insecticides to reduce the frequency of application and delay further evolution of resistance. Field trials of spirotetramat against cowpea thrips demonstrated low control efficacy (Table 5), which may be attributed to the spray treatment of insecticides against thrips. Thigmotactic behavior in flowers shields thrips from contact with foliar spays [42]. Although a flower injection of spirotetramat has been proposed as an effective method for banana thrips control [42], this technique is clearly impractical for cowpea. Alternatively, drip irrigation of systemic insecticides in the seedling stage of cowpea may present a viable strategy [43].

Laboratory toxicity assays of insecticides provide only partial insights into their pest control potential, as field efficacy is subject to dynamic interactions between biotic and abiotic factors. Therefore, we chose five commonly used insecticides to evaluate their field control efficacy against *M. usitatus* in a farm located in Yunfu. Among these insecticides, spinetoram showed good toxicity in both laboratory and field experiments, with an LC_50_ between approximately 0.1 and 0.7 and corrected control efficacy of 43~75% within a week after application (Table 2 and Table 5). Previous research also suggested spinetoram as a useful tool for thrips management due to its efficacy and selectivity for certain pest species and conservation of natural enemies [44,45]. Though the laboratory toxicity of cyantraniliprole against *M. usitatus* field populations was not the highest, it showed the best control efficacy when applied in the field, with a corrected control efficacy of 76% (Table 5). Field experiments showed that cyantraniliprole significantly reduced the population of sucking pests, and the relative efficacy against *thrips tabaci* was over 80% [46], which was in line with our field experiments against *M. usitatus.* Nonetheless, spirotetramat showed the lowest toxicity but not the least efficient insecticides (with corrected control efficacy of 60% at 7 dpa) when applied in the field. In summary, cyantraniliprole, spinetoram and emamectin benzoate emerged as optimal candidates for field deployment against *M. usitatus*. Intriguingly, they generated thrips resistance against several chemicals, which are still effective when applied in the field, such as emamectin benzoate (Table 4 and Table 5). For one aspect, the extremely low baseline susceptibility of the reference strain (LC_50_ = 0.006 mg a.i./L) [25] artificially inflated the RR values, potentially overestimating resistance severity. For another aspect, the field-recommended dosage of emamectin benzoate was 2.625–3.375 g a.i./l, while the LC_50_ of the three populations was 0.59–17.87 a.i. mg/l, which was far less than the recommended dosage and might explain its high efficacy when applied in the field.

## 5. Conclusions

This study evaluated the toxicity of 10 insecticides against *M. usitatus* populations from three regions in Guangdong, China. Laboratory bioassays revealed significant regional variations in susceptibility: spinetoram and spinosad exhibited consistently high toxicity across all populations, while resistance to avermectin, chlorfenapyr, and spirotetramat was notably elevated. Field trials confirmed spinetoram and cyantraniliprole as effective options, achieving over 75% control efficacy at 7 days post-application. The findings advocate for integrating insecticides with diverse modes of action (e.g., broflanilide, emamectin benzoate) into rotation strategies to mitigate resistance evolution and ensure sustainable thrips control in cowpea production systems.

## Figures and Tables

**Table 1:** Laboratory toxicity of 10 insecticides against QY population of *M. usitatus*.

Field Population	Insecticide	Number of Tested Thrips	LC_50_ (mg a.i./L) (95% Confidence Interval)	Slope (SE)	C^2^	*Df*	*p*
QY	broflanilide	335	7.40 (4.45–11.93)	0.71 ± 0.09	1.52	4	0.82
	dinotefuran	329	56.884 (35.732–90.489)	0.70 ± 0.08	0.81	4	0.94
	spinetoram	286	0.14 (0.03–0.41)	1.00 ± 0.13	7.13	5	0.07
	spinosad	264	0.27 (0.10–0.73)	0.91 ± 0.11	4.89	3	0.18
	cyantraniliprole	321	60.77 (15.81–187.39)	0.76 ± 0.11	7.02	4	0.13
	spirotetramat	334	987.80 (363.00–1686.11)	2.67 ± 0.42	8.83	4	0.07
	emamectin benzoate	386	17.87 (6.29–43.80)	0.87 ± 0.09	10.58	5	0.06
	avermectin	276	43.31 (18.96–80.42)	0.71 ± 0.10	2.45	3	0.48
	thiamethoxam	383	174.91 (60.15–292.15)	1.83 ± 0.31	7.55	5	0.18
	chlorfenapyr	283	36.35 (12.06–102.01)	0.70 ± 0.10	10.45	5	0.06

**Table 2:** Laboratory toxicity of 10 insecticides against YF population of *M. usitatus*.

Field Population	Insecticide	Number of Tested Thrips	LC_50_ (mg a.i./L) (95% Confidence Interval)	Slope (SE)	C^2^	*Df*	*p*
YF	broflanilide	253	13.93 (6.40–25.26)	0.73 ± 0.12	2.11	4	0.72
	dinotefuran	328	112.80 (29.16–326.25)	0.77 ± 0.16	5.56	5	0.35
	spinetoram	420	0.67 (0.20–1.12)	3.68 ± 0.70	10.20	5	0.07
	spinosad	424	2.03 (1.16–3.03)	4.20 ± 0.78	6.911	5	0.23
	cyantraniliprole	415	35.67 (21.54–51.06)	1.97 ± 0.35	1.84	5	0.87
	spirotetramat	362	1237.15 (605.54–1752.98)	2.86 ± 0.59	5.47	5	0.36
	emamectin benzoate	361	0.59 (0.14–1.55)	0.88 ± 0.10	8.74	4	0.07
	avermectin	385	675.73 (341.04–1843.43)	0.70 ± 0.12	3.63	5	0.60
	thiamethoxam	382	103.97 (29.04–202.76)	1.43 ± 0.21	8.94	5	0.11
	chlorfenapyr	360	122.28 (59.81–222.18)	0.99 ± 0.18	2.70	5	0.75

**Table 3:** Laboratory toxicity of 10 insecticides against MM population of *M. usitatus*.

Field Population	Insecticide	Number of Tested Thrips	LC_50_ (mg a.i./L) (95% Confidence Interval)	Slope (SE)	C^2^	*Df*	*p*
MM	broflanilide	264	71.33 (38.40~126.72)	0.72 ± 0.12	1.63	5	0.90
	dinotefuran	232	13.24 (2.41~39.41)	0.70 ± 0.10	6.01	4	0.20
	spinetoram	327	0.26 (0.17~0.39)	1.47 ± 0.19	2.31	5	0.81
	spinosad	343	0.40 (0.24~0.59)	1.35 ± 0.18	2.63	5	0.76
	cyantraniliprole	216	11.02 (4.47~20.17)	0.75 ± 0.14	0.73	3	0.40
	spirotetramat	286	113.19 (62.53~178.25)	1.30 ± 0.18	2.94	5	0.71
	emamectin benzoate	199	4.82 (0.65~14.17)	0.74 ± 0.13	3.49	3	0.32
	avermectin	208	360.80 (68.94~989.34)	0.78 ± 0.16	3.19	3	0.36
	thiamethoxam	285	15.74 (2.81~40.69)	1.00 ± 0.14	6.34	4	0.17
	chlorfenapyr	243	19.08 (8.41~34.15)	1.19 ± 0.15	5.32	5	0.38

**Table 4:** Resistance level of different *M. usitatus* field strains against 6 insecticides.

Insecticide	LC_50_ (95% Confidence Interval)	Resistance Ratio (RR)
spinetoram	0.017 (0.010–0.028) Susceptible strain ^a^	8.47 (QY)
		39.47 (YF)
		15.53 (MM)
emamectin benzoate	0.006 (0.004–0.011) Susceptible strain ^a^	2978 (QY)
		98.50 (YF)
		802.67 (MM)
avermectin	0.276 (0.129–0.594) Susceptible strain ^a^	156.92 (QY)
		2448.30 (YF)
		1307.25 (MM)
spirotetramat	3.143 (1.748–5.650) Susceptible strain ^a^	314.29 (QY)
		939.62 (YF)
		36.01 (MM)
spinosad	0.172 (0.091–0.249) Susceptible strain ^b^	1.59 (QY)
		11.80 (YF)
		2.30 (MM)
chlorfenapyr	0.268 (0.155–0.462) Susceptible strain ^a^	135.63 (QY)
		456.27 (YF)
		79.19 (MM)

^a^ Data adopted from previous research [25]; ^b^ Data adopted from previous research [24]. Insecticide bioassay method used in these references were “leaf-tube residual film method”, which was consistent with method used in our study.

**Table 5:** Field control efficacy of five insecticides against *M. usitatus*.

Insecticides	Dosage (g a.i./ha)	Initial Number (10 Plants)	Number of Thrips (10 Plants)				Reduction Rate (%)				Corrected Control Efficacy (%)			
			1 dpa	3 dpa	5 dpa	7 dpa	1 dpa	3 dpa	5 dpa	7 dpa	1 dpa	3 dpa	5 dpa	7 dpa
cyantraniliprole	60	100	56	50	42	28	44.00	50.25	57.75	71.75	52.58 a	59.98 a	61.08 a	76.35 a
spirotetramat	100.8	86	71	45	41	41	17.39	48.12	52.46	52.46	30.05 a	58.27 a	56.21 a	60.21 ab
emamectin benzoate	3.375	104	64	62	44	37.5	38.65	40.58	57.25	63.77	48.05 a	52.20 a	60.62 a	69.67 ab
thiamethoxam	75	105	74	78	50	52	29.12	26.01	52.51	50.36	39.98 a	40.49 a	56.25 a	58.44 b
spinetoram	18	98	66	59	49	29	32.91	39.54	50.26	70.41	43.19 a	51.37 a	54.18 a	75.23 a
Water control	-	129	152	160	140	154	−18.09	−24.32	−8.56	−19.46	-	-	-	-

Differet letter indicates significant difference, *p* < 0.05.

## Data Availability

The original contributions presented in this study are included in the article; further inquiries can be directed to the corresponding authors.

## Supplementary Materials

The following supporting information can be downloaded at: https://www.mdpi.com/article/10.3390/insects16050459/s1, Figure S1: Map of Guangdong province showing the *M. usitatus* collection sites; Figure S2: Schematic drawing of the experimental layout for field experiment; Table S1: Recommended field doses of four used insecticides; Table S2: Concentration gradients of insecticides against QY population; Table S3: Concentration gradients of insecticides against YF; Table S4: Concentration gradients of insecticides against MM population.
