# Supplementary material for: Field-Based Evaluation of Insecticide Effectiveness on Megalurothrips usitatus in Guangdong, China: Implications for Pest Control Strategies
Source: Insects. 2025 Apr 27;16(5):459. doi: 10.3390/insects16050459 (PMC12112036; doi:10.3390/insects16050459)
Supplement: Supplementary file 1 [file insects-16-00459-s001.zip › insects-3533946-supplementary.pdf]

Table S1. Recommended field doses of four used insecticides.

**Insecticide**	**Active ingredient (AI)**	**Recommended field doses**	**Water (kg/ha)**
Broflanilide	100 g/L	195-240 ml/ha	900
Dinotefuran	20%	450-600 ml/ha	750
Spinetoram	60 g/L	600-750 ml/ha	900
Spinosad	10%	300-375 ml/ha	900
Cyantraniliprole	10%	600-750 ml/ha	900
Spirotetramat	22.4%	375-450 ml/ha	900
Emamectin benzoate	5%	52.5-67.5 g/ha	900
Avermectin	1.8%	1200-600 ml/ha	900
Thiamethoxam	25%	225-300 ml/ha	900
Chlorfenapyr	100 g/L	720-1080 ml/ha	750

Table S2 Concentration gradients of insecticides against QY population

Insecticides	Concentration gradients (mg a.i. /l)					
broflanilide	0.24		3.90625	15.625	62.5	250	1000
dinotefuran	0.15625	0.625	2.5	10	40	160	640
spinetoram	0.0015	0.0059	0.023	0.09375	0.375	1.5	6
spinosad	0.0049	0.02	0.78125	0.3125	1.25	5	20
cyantraniliprole	0.53	2.11	8.4375	33.75	135	540	2160
spirotetramat	15.36	46.09	138.27	414.81	1244.44	3733.33	11200
emamectin benzoate	0.0625	0.25	0.75	3	12	48	192
avermectin	1.372	4.115	12.346	37.037	111.11	333.33	1000
thiamethoxam							
chlorfenapyr	0.37	1.46	5.86	23.4375	93.75	375	1500

Table S3 Concentration gradients of insecticides against YF population

Insecticides	Concentration gradients (mg a.i. /l)						
broflanilide	0.24	0.98	3.91	15.625	62.5		1000
dinotefuran	0.24	0.98		15.625	62.5	250	1000
spinetoram	0.0015	0.0059		0.09375		1.5	6
spinosad	0.0049	0.02	0.078125		1.25		20
cyantraniliprole		0.98	3.91	15.625	62.5	250	1000
spirotetramat	15.36	46.09		414.81	1244.44	3733.33	11200
emamectin benzoate	0.25	0.99	3.95	15.8125	63.25	253	1012
avermectin	1.22			78.125	312.5	1250	5000
thiamethoxam	6.86	20.58	61.73	185.19	555.56	1666.67	5000
chlorfenapyr	0.24	0.98	3.91	15.625	62.5	250	1000

Table S4 Concentration gradients of insecticides against MM population

Insecticides	Concentration gradients (mg a.i. /l)						
broflanilide	1.37	4.12	12.35	37.034	111.11	333.33	1000
dinotefuran	2.44	9.766		156.25	625	2500	10000
spinetoram	0.005859	0.023438	0.09375	0.375	1.5	6	24
spinosad	0.00488	0.0195	0.078125	0.3125	1.25	5	20
cyantraniliprole			3.91	15.625	62.5	250	1000
spirotetramat	15.36	46.09	138.27	414.81	1244.44	3733.33	11200
emamectin benzoate	0.25	0.99		15.8125	63.25	253	1012
avermectin	1.22	4.88		78.125		1250	5000
thiamethoxam		2.93	11.75	46.875	187.5	750	3000
chlorfenapyr	2.06	6.17	18.52	55.56	166.67	500	1500

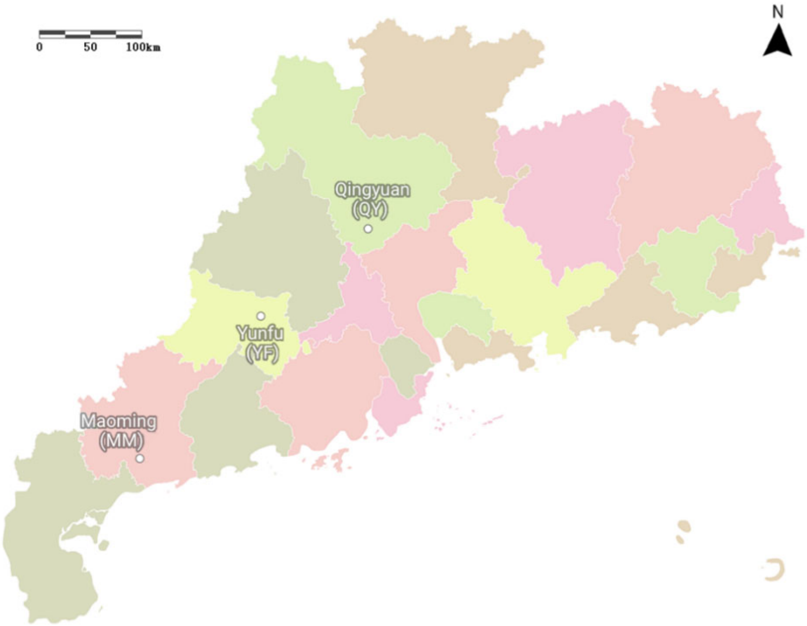

Figure S1 Map of Guangdong province showing the *M. usitatus* collection sites. Supervised by Guangdong Administration of Surveying Mapping and Geoinformation.

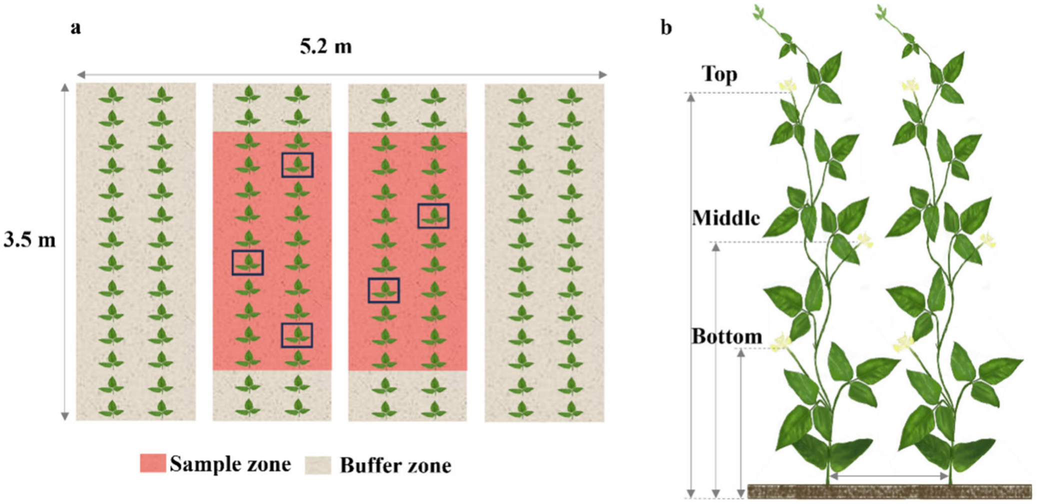

Figure S2 Schematic drawing of the experimental layout for field experiment. (a) Represents a testing plot, including both the sample zone (treated) and marginal buffer zones (untreated) surrounding each side of the sample zone. (b) Illustrates sampling sites of each randomly selected cowpea plant.
